# Bis(phenyl­phospho­nic) anhydride

**DOI:** 10.1107/S1600536809038525

**Published:** 2009-10-03

**Authors:** Yang Li, Guoxiong Hua, Alexandra M. Z. Slawin, J. Derek Woollins

**Affiliations:** a Department of Chemistry, University of St Andrews, Fife KY16 9ST, Scotland

## Abstract

The asymmetric unit of the title compound, C_12_H_12_O_5_P_2_, contains four independent mol­ecules, generating two dimers *via* pairs of inter­molecular O—H⋯O hydrogen bonds, forming *R*_2_^2^(8) rings. The two aryl rings of each mol­ecule form dihedral angles of 108.6 (1), 103.2 (1), 12.5 (2) and 8.1 (2)° in the four mol­ecules.

## Related literature

For related structural information, see: Kingsley *et al.* (2001 ▶); Bernstein *et al.* (1995 ▶). For syntheses, see: Ruveda *et al.* (1973 ▶); Gallagher & Jenkins (1966 ▶); Mikolajczyk (1966 ▶).
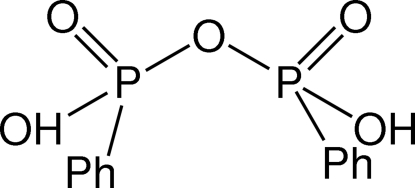


## Experimental

### 

#### Crystal data

C_12_H_12_O_5_P_2_
*M* *_r_* = 298.16
Triclinic, 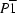

*a* = 5.6510 (7) Å
*b* = 19.3320 (18) Å
*c* = 24.440 (3) Å
α = 84.701 (8)°
β = 89.192 (8)°
γ = 81.687 (7)°
*V* = 2630.6 (5) Å^3^
*Z* = 8
Mo *K*α radiation
μ = 0.34 mm^−1^
*T* = 93 K
0.05 × 0.05 × 0.03 mm

#### Data collection

Rigaku Mercury CCD diffractometer
Absorption correction: multi-scan (*CrystalClear*; Rigaku, 2004 ▶) *T*_min_ = 0.983, *T*_max_ = 0.990
18800 measured reflections
9791 independent reflections
6873 reflections with *I* > 2σ(*I*)
*R*_int_ = 0.052

#### Refinement

*R*[*F* ^2^ > 2σ(*F* ^2^)] = 0.054
*wR*(*F* ^2^) = 0.142
*S* = 1.07
9791 reflections
694 parameters
H-atom parameters constrained
Δρ_max_ = 0.69 e Å^−3^
Δρ_min_ = −0.50 e Å^−3^

### 

Data collection: *CrystalClear* (Rigaku, 2004 ▶); cell refinement: *CrystalClear*; data reduction: *CrystalClear*; program(s) used to solve structure: *SHELXS97* (Sheldrick, 2008 ▶); program(s) used to refine structure: *SHELXL97* (Sheldrick, 2008 ▶); molecular graphics: *PLATON* (Spek, 2009 ▶); software used to prepare material for publication: *SHELXTL* (Sheldrick, 2008 ▶).

## Supplementary Material

Crystal structure: contains datablocks I, global. DOI: 10.1107/S1600536809038525/pk2185sup1.cif

Structure factors: contains datablocks I. DOI: 10.1107/S1600536809038525/pk2185Isup2.hkl

Additional supplementary materials:  crystallographic information; 3D view; checkCIF report

## Figures and Tables

**Table 1:** Hydrogen-bond geometry (Å, °)

*D*—H⋯*A*	*D*—H	H⋯*A*	*D*⋯*A*	*D*—H⋯*A*
O41—H41⋯O45^i^	0.84	1.69	2.530 (3)	175
O64—H64⋯O62^ii^	0.84	1.67	2.488 (3)	163
O61—H61⋯O65^iii^	0.84	1.72	2.551 (3)	171
O4—H4⋯O25	0.84	1.69	2.464 (3)	153
O21—H21⋯O2	0.84	1.70	2.472 (3)	152
O24—H24⋯O22^i^	0.84	1.67	2.464 (3)	156
O1—H1⋯O5^iii^	0.84	1.66	2.456 (3)	157

Symmetry codes: (i) 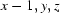; (ii) 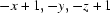; (iii) 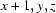.

**Fig. 1.**
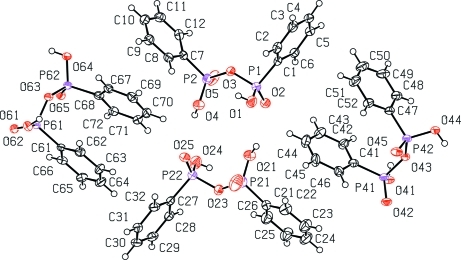
The molecular structure of the title compound with displacement ellipsoids drawn at the 50% probability level.

## supplementary crystallographic information

### Comment

P^1^P^2^-disubstituted pyrophosphoric acid has been prepared by the reaction of organophosphorus(V) dichlorides, organophosphorus(III) dichlorides and organothiophosphoryl dichlorides with dimethylsulphoxide (DMSO) (Ruveda *et al*., 1973; Mikolajczyk, 1966). We report here the synthesis of the title compound by the reaction of succinyl chloride with Woollins' reagent. The *x*-ray structure reveals that the title compound exists as an independent molecule rather than as a part (mono-anion or anion dimer) of a molecule which has been reported in the literature (Kingsley *et al.*, 2001).

The molecular structure of the title compound is shown in Fig. 1. In the crystal the molecules stacks up the *a* axis and are connected to one another *via* pairs of intermolecular O—H···O hydrogen bonds, with *R*^2^_2_(8) motif (Bernstein *et al.*, 1995), forming two types of dimers [Table 1]. The four independent molecules have two types of conformations with different dihedral angles between the two benzene rings as described in the abstract.

### Experimental

A mixture of succinyl chloride (0.16 g, 1 mmol) and Woollins' reagent (0.27 g, 0.5 mmol) in dry toluene (5 ml) was refluxed for 6 hr. Upon cooling to room temperature the mixture was exposed in the air overnight and purified by silica gel (toluene as eluent) to give diphenyldiphosphonic acid (white paste, 0.168 g, 56%). Colourless crystal was obtained by slow evaporation of chloromethane solution.

### Refinement

All H atoms were fixed geometrically (C—H = 0.95 Å, O—H = 0.84 Å) and treated as riding with *U*_iso_(H) = 1.2Ueq(C) or *U*_iso_(H) = 1.5Ueq(O) of the parent atom.

### Crystal data

**Table:**

C_12_H_12_O_5_P_2_	*Z* = 8
*M**_r_* = 298.16	*F*(000) = 1232
Triclinic, *P*1	*D*_x_ = 1.506 Mg m^−^^3^
Hall symbol: -P 1	Mo *K*α radiation, λ = 0.71073 Å
*a* = 5.6510 (7) Å	Cell parameters from 8791 reflections
*b* = 19.3320 (18) Å	θ = 1.7–28.5°
*c* = 24.440 (3) Å	µ = 0.34 mm^−^^1^
α = 84.701 (8)°	*T* = 93 K
β = 89.192 (8)°	Prism, colourless
γ = 81.687 (7)°	0.05 × 0.05 × 0.03 mm
*V* = 2630.6 (5) Å^3^	

### Data collection

**Table:**

Rigaku Mercury CCD diffractometer	9791 independent reflections
Radiation source: rotating anode	6873 reflections with *I* > 2σ(*I*)
confocal multilayer optics	*R*_int_ = 0.052
Detector resolution: 0.83 pixels mm^-1^	θ_max_ = 28.5°, θ_min_ = 1.3°
ω scans	*h* = −7→5
Absorption correction: multi-scan (*CrystalClear*; Rigaku, 2004)	*k* = −24→25
*T*_min_ = 0.983, *T*_max_ = 0.990	*l* = −31→31
18800 measured reflections	

### Refinement

**Table:**

Refinement on *F*^2^	Primary atom site location: structure-invariant direct methods
Least-squares matrix: full	Secondary atom site location: difference Fourier map
*R*[*F*^2^ > 2σ(*F*^2^)] = 0.054	Hydrogen site location: inferred from neighbouring sites
*wR*(*F*^2^) = 0.142	H-atom parameters constrained
*S* = 1.07	*w* = 1/[σ^2^(*F*_o_^2^) + (0.0599*P*)^2^] where *P* = (*F*_o_^2^ + 2*F*_c_^2^)/3
9791 reflections	(Δ/σ)_max_ = 0.034
694 parameters	Δρ_max_ = 0.69 e Å^−^^3^
0 restraints	Δρ_min_ = −0.50 e Å^−^^3^

### Special details

**Table:**

Geometry. All e.s.d.'s (except the e.s.d. in the dihedral angle between two l.s. planes) are estimated using the full covariance matrix. The cell e.s.d.'s are taken into account individually in the estimation of e.s.d.'s in distances, angles and torsion angles; correlations between e.s.d.'s in cell parameters are only used when they are defined by crystal symmetry. An approximate (isotropic) treatment of cell e.s.d.'s is used for estimating e.s.d.'s involving l.s. planes.
Refinement. Refinement of *F*^2^ against ALL reflections. The weighted *R*-factor *wR* and goodness of fit *S* are based on *F*^2^, conventional *R*-factors *R* are based on *F*, with *F* set to zero for negative *F*^2^. The threshold expression of *F*^2^ > 2σ(*F*^2^) is used only for calculating *R*-factors(gt) *etc*. and is not relevant to the choice of reflections for refinement. *R*-factors based on *F*^2^ are statistically about twice as large as those based on *F*, and *R*- factors based on ALL data will be even larger.

### Fractional atomic coordinates and isotropic or equivalent isotropic displacement parameters (Å^2^)

**Table:**

	*x*	*y*	*z*	*U*_iso_*/*U*_eq_	
P1	1.16675 (16)	0.61566 (4)	0.30579 (3)	0.0279 (2)	
P2	0.97193 (17)	0.49073 (4)	0.35149 (3)	0.0290 (2)	
P21	1.01976 (17)	0.51094 (4)	0.15068 (4)	0.0331 (2)	
P22	0.82971 (16)	0.38450 (4)	0.19407 (3)	0.0287 (2)	
P41	0.27280 (14)	0.88570 (4)	0.03348 (3)	0.02070 (19)	
P42	0.48620 (14)	0.97558 (4)	0.10244 (3)	0.02159 (19)	
P62	0.45738 (14)	0.12067 (4)	0.45418 (3)	0.01997 (19)	
P61	0.77543 (14)	0.00887 (4)	0.40704 (3)	0.01895 (19)	
O41	0.0099 (4)	0.89512 (11)	0.01611 (8)	0.0264 (5)	
H41	−0.0768	0.9111	0.0416	0.040*	
O64	0.4511 (4)	0.13924 (10)	0.51383 (8)	0.0263 (5)	
H64	0.4316	0.1037	0.5349	0.039*	
O63	0.6907 (3)	0.06224 (9)	0.45239 (8)	0.0195 (4)	
O43	0.2930 (4)	0.95729 (9)	0.06046 (8)	0.0216 (5)	
O45	0.7276 (4)	0.93941 (10)	0.09058 (8)	0.0281 (5)	
O65	0.2455 (4)	0.09410 (10)	0.43377 (8)	0.0253 (5)	
O61	1.0438 (4)	−0.01264 (10)	0.41936 (9)	0.0234 (5)	
H61	1.1032	0.0225	0.4274	0.035*	
O62	0.6400 (4)	−0.05156 (9)	0.41066 (8)	0.0230 (5)	
O44	0.4503 (4)	1.05595 (10)	0.09769 (8)	0.0287 (5)	
H44	0.4761	1.0712	0.0652	0.043*	
O42	0.4414 (4)	0.87522 (10)	−0.01302 (8)	0.0260 (5)	
O4	1.1294 (5)	0.45037 (12)	0.30940 (9)	0.0406 (6)	
H4	1.0423	0.4322	0.2889	0.061*	
O5	0.7134 (5)	0.50673 (13)	0.34033 (11)	0.0502 (7)	
O2	0.9874 (4)	0.62696 (11)	0.26044 (9)	0.0357 (6)	
O22	1.2784 (5)	0.49518 (14)	0.16155 (11)	0.0569 (8)	
O3	1.0983 (5)	0.55921 (10)	0.35369 (9)	0.0358 (6)	
O21	0.8651 (5)	0.54977 (12)	0.19411 (10)	0.0442 (7)	
H21	0.9486	0.5738	0.2106	0.066*	
O23	0.8914 (5)	0.44308 (10)	0.14710 (9)	0.0383 (6)	
O25	0.9972 (4)	0.37785 (11)	0.24119 (9)	0.0344 (6)	
O24	0.5660 (4)	0.40140 (12)	0.20872 (11)	0.0446 (7)	
H24	0.4994	0.4333	0.1860	0.067*	
O1	1.4214 (4)	0.59276 (12)	0.28741 (10)	0.0406 (6)	
H1	1.4907	0.5626	0.3111	0.061*	
C46	0.5533 (6)	0.77233 (15)	0.08698 (14)	0.0292 (7)	
H46	0.6681	0.7794	0.0590	0.035*	
C66	0.5408 (6)	0.05112 (15)	0.30895 (12)	0.0250 (7)	
H66	0.4325	0.0195	0.3209	0.030*	
C67	0.5304 (5)	0.19544 (14)	0.41378 (12)	0.0223 (7)	
C48	0.1545 (6)	0.97859 (16)	0.18590 (13)	0.0283 (7)	
H48	0.0560	1.0120	0.1620	0.034*	
C42	0.1633 (6)	0.80378 (16)	0.12647 (13)	0.0298 (8)	
H42	0.0122	0.8327	0.1258	0.036*	
C41	0.3325 (6)	0.81587 (15)	0.08616 (12)	0.0239 (7)	
C72	0.3964 (6)	0.22236 (15)	0.36675 (12)	0.0274 (7)	
H72	0.2618	0.2017	0.3572	0.033*	
C47	0.3815 (6)	0.94968 (15)	0.16860 (12)	0.0230 (7)	
C65	0.5087 (6)	0.09054 (16)	0.25850 (12)	0.0279 (7)	
H65	0.3785	0.0856	0.2357	0.033*	
C62	0.8901 (6)	0.10533 (15)	0.32413 (12)	0.0256 (7)	
H62	1.0215	0.1103	0.3466	0.031*	
C61	0.7339 (5)	0.05836 (13)	0.34192 (11)	0.0185 (6)	
C68	0.7283 (6)	0.22652 (14)	0.42730 (13)	0.0262 (7)	
H68	0.8193	0.2088	0.4593	0.031*	
C63	0.8551 (6)	0.14466 (16)	0.27425 (13)	0.0286 (7)	
H63	0.9611	0.1770	0.2625	0.034*	
C49	0.0736 (7)	0.95862 (18)	0.23771 (13)	0.0359 (8)	
H49	−0.0798	0.9788	0.2495	0.043*	
C7	1.0219 (6)	0.44602 (14)	0.41789 (12)	0.0245 (7)	
C21`	0.9653 (6)	0.55748 (15)	0.08425 (13)	0.0285 (7)	
C6	0.9476 (6)	0.74039 (15)	0.34043 (12)	0.0263 (7)	
H6	0.8195	0.7351	0.3171	0.032*	
C9	1.2736 (6)	0.36786 (16)	0.48229 (13)	0.0297 (8)	
H9	1.4227	0.3398	0.4913	0.036*	
C28	0.6996 (6)	0.29289 (16)	0.12371 (12)	0.0276 (7)	
H28	0.5529	0.3238	0.1193	0.033*	
C31	1.1277 (6)	0.20238 (16)	0.13638 (13)	0.0304 (8)	
H31	1.2745	0.1715	0.1405	0.037*	
C70	0.6554 (7)	0.30892 (16)	0.34780 (14)	0.0339 (8)	
H70	0.6984	0.3479	0.3253	0.041*	
C5	0.9271 (6)	0.79708 (16)	0.37234 (13)	0.0300 (8)	
H5	0.7855	0.8303	0.3712	0.036*	
C51	0.4381 (7)	0.88049 (19)	0.25558 (14)	0.0413 (9)	
H51	0.5343	0.8468	0.2797	0.050*	
C45	0.6035 (7)	0.71889 (16)	0.12873 (15)	0.0372 (9)	
H45	0.7548	0.6901	0.1299	0.045*	
C10	1.0901 (6)	0.37050 (16)	0.52002 (13)	0.0313 (8)	
H10	1.1127	0.3443	0.5549	0.038*	
C71	0.4614 (7)	0.27951 (15)	0.33401 (13)	0.0318 (8)	
H71	0.3709	0.2980	0.3021	0.038*	
C43	0.2155 (6)	0.74958 (17)	0.16757 (14)	0.0363 (8)	
H43	0.1003	0.7413	0.1952	0.044*	
C50	0.2131 (8)	0.91000 (19)	0.27211 (14)	0.0433 (10)	
H50	0.1554	0.8963	0.3076	0.052*	
C11	0.8710 (6)	0.41162 (16)	0.50708 (14)	0.0313 (8)	
H11	0.7451	0.4138	0.5333	0.038*	
C52	0.5222 (6)	0.90029 (16)	0.20367 (13)	0.0309 (8)	
H52	0.6759	0.8800	0.1922	0.037*	
C64	0.6649 (6)	0.13691 (17)	0.24123 (13)	0.0313 (8)	
H64A	0.6418	0.1636	0.2066	0.038*	
C27	0.8736 (6)	0.30733 (15)	0.15891 (12)	0.0244 (7)	
C30	0.9528 (7)	0.18844 (17)	0.10142 (13)	0.0351 (8)	
H30	0.9794	0.1478	0.0818	0.042*	
C32	1.0882 (6)	0.26151 (16)	0.16542 (13)	0.0289 (7)	
H32	1.2073	0.2707	0.1897	0.035*	
C25	0.7334 (7)	0.58889 (19)	0.00260 (15)	0.0468 (10)	
H25	0.6037	0.5827	−0.0199	0.056*	
C24	0.8800 (7)	0.6373 (2)	−0.01487 (15)	0.0454 (10)	
H24A	0.8506	0.6649	−0.0490	0.054*	
C12	0.8367 (6)	0.44912 (15)	0.45645 (13)	0.0286 (7)	
H12	0.6873	0.4771	0.4477	0.034*	
C69	0.7911 (6)	0.28281 (15)	0.39421 (14)	0.0312 (8)	
H69	0.9264	0.3036	0.4031	0.037*	
C2	1.3433 (6)	0.69980 (16)	0.37591 (13)	0.0300 (8)	
H2	1.4849	0.6666	0.3773	0.036*	
C1	1.1543 (6)	0.69148 (14)	0.34250 (12)	0.0251 (7)	
C29	0.7391 (7)	0.23369 (17)	0.09511 (13)	0.0325 (8)	
H29	0.6195	0.2241	0.0711	0.039*	
C3	1.3254 (7)	0.75645 (16)	0.40719 (13)	0.0333 (8)	
H3	1.4557	0.7625	0.4295	0.040*	
C8	1.2425 (6)	0.40591 (15)	0.43110 (13)	0.0275 (7)	
H8	1.3704	0.4046	0.4054	0.033*	
C22	1.1138 (7)	0.60564 (18)	0.06687 (14)	0.0377 (8)	
H22	1.2459	0.6112	0.0889	0.045*	
C4	1.1176 (7)	0.80421 (16)	0.40597 (13)	0.0342 (8)	
H4A	1.1047	0.8423	0.4283	0.041*	
C26	0.7717 (7)	0.54935 (18)	0.05210 (15)	0.0424 (9)	
H26	0.6671	0.5168	0.0643	0.051*	
C44	0.4361 (7)	0.70732 (17)	0.16835 (15)	0.0381 (9)	
H44A	0.4713	0.6701	0.1965	0.046*	
C23	1.0706 (7)	0.64575 (19)	0.01752 (16)	0.0466 (10)	
H23	1.1722	0.6793	0.0057	0.056*	

### Atomic displacement parameters (Å^2^)

**Table:**

	*U*^11^	*U*^22^	*U*^33^	*U*^12^	*U*^13^	*U*^23^
P1	0.0377 (5)	0.0226 (4)	0.0237 (4)	−0.0054 (4)	0.0035 (4)	−0.0026 (3)
P2	0.0403 (5)	0.0235 (4)	0.0237 (4)	−0.0074 (4)	−0.0037 (4)	0.0007 (3)
P21	0.0417 (6)	0.0279 (4)	0.0294 (5)	−0.0055 (4)	−0.0065 (4)	0.0009 (4)
P22	0.0387 (5)	0.0236 (4)	0.0239 (4)	−0.0044 (4)	0.0016 (4)	−0.0032 (3)
P41	0.0231 (4)	0.0235 (4)	0.0162 (4)	−0.0043 (3)	0.0033 (3)	−0.0040 (3)
P42	0.0244 (4)	0.0244 (4)	0.0172 (4)	−0.0058 (3)	0.0048 (3)	−0.0056 (3)
P62	0.0229 (4)	0.0200 (4)	0.0183 (4)	−0.0059 (3)	0.0032 (3)	−0.0046 (3)
P61	0.0216 (4)	0.0195 (4)	0.0168 (4)	−0.0051 (3)	0.0019 (3)	−0.0040 (3)
O41	0.0235 (12)	0.0378 (12)	0.0190 (11)	−0.0036 (10)	0.0042 (9)	−0.0095 (9)
O64	0.0400 (14)	0.0227 (10)	0.0185 (11)	−0.0105 (10)	0.0083 (10)	−0.0065 (8)
O63	0.0216 (11)	0.0214 (10)	0.0162 (10)	−0.0035 (8)	0.0010 (9)	−0.0048 (8)
O43	0.0249 (11)	0.0224 (10)	0.0171 (10)	−0.0005 (9)	0.0000 (9)	−0.0038 (8)
O45	0.0238 (12)	0.0365 (12)	0.0247 (12)	−0.0029 (10)	0.0047 (10)	−0.0093 (9)
O65	0.0246 (12)	0.0235 (10)	0.0299 (12)	−0.0087 (9)	0.0019 (10)	−0.0057 (9)
O61	0.0214 (11)	0.0209 (10)	0.0281 (12)	−0.0032 (9)	−0.0001 (10)	−0.0042 (9)
O62	0.0280 (12)	0.0217 (10)	0.0209 (11)	−0.0089 (9)	0.0018 (9)	−0.0028 (8)
O44	0.0443 (14)	0.0229 (10)	0.0198 (11)	−0.0070 (10)	0.0105 (11)	−0.0047 (9)
O42	0.0294 (12)	0.0305 (11)	0.0192 (11)	−0.0063 (9)	0.0085 (10)	−0.0068 (9)
O4	0.0607 (17)	0.0352 (13)	0.0265 (13)	−0.0060 (12)	−0.0034 (12)	−0.0066 (10)
O5	0.0450 (16)	0.0572 (16)	0.0453 (16)	−0.0100 (13)	−0.0085 (14)	0.0170 (13)
O2	0.0553 (16)	0.0278 (11)	0.0262 (12)	−0.0121 (11)	−0.0001 (12)	−0.0047 (9)
O22	0.0413 (16)	0.0653 (18)	0.0592 (19)	−0.0051 (14)	−0.0119 (15)	0.0176 (15)
O3	0.0637 (17)	0.0244 (11)	0.0213 (12)	−0.0140 (11)	0.0011 (12)	−0.0011 (9)
O21	0.0661 (19)	0.0387 (14)	0.0288 (13)	−0.0064 (13)	−0.0068 (13)	−0.0085 (11)
O23	0.0657 (18)	0.0259 (11)	0.0246 (12)	−0.0122 (11)	−0.0035 (12)	0.0001 (9)
O25	0.0498 (15)	0.0296 (11)	0.0257 (12)	−0.0090 (11)	0.0002 (11)	−0.0082 (9)
O24	0.0411 (15)	0.0400 (14)	0.0462 (16)	0.0087 (12)	0.0074 (13)	0.0071 (12)
O1	0.0449 (15)	0.0344 (13)	0.0363 (14)	0.0090 (11)	0.0081 (12)	0.0053 (11)
C46	0.0295 (18)	0.0242 (16)	0.0339 (19)	−0.0038 (14)	0.0072 (16)	−0.0038 (14)
C66	0.0250 (17)	0.0285 (16)	0.0227 (16)	−0.0035 (13)	0.0023 (14)	−0.0092 (13)
C67	0.0217 (16)	0.0264 (15)	0.0212 (16)	−0.0089 (13)	0.0048 (13)	−0.0071 (13)
C48	0.0305 (18)	0.0322 (16)	0.0239 (17)	−0.0082 (14)	0.0044 (15)	−0.0063 (13)
C42	0.0291 (18)	0.0330 (17)	0.0262 (17)	−0.0039 (14)	0.0052 (15)	0.0026 (14)
C41	0.0269 (17)	0.0271 (15)	0.0194 (15)	−0.0074 (13)	0.0032 (14)	−0.0058 (13)
C72	0.0309 (18)	0.0265 (15)	0.0254 (17)	−0.0041 (14)	0.0023 (15)	−0.0061 (13)
C47	0.0264 (17)	0.0273 (16)	0.0168 (15)	−0.0063 (13)	0.0003 (14)	−0.0053 (13)
C65	0.0284 (18)	0.0359 (17)	0.0201 (16)	−0.0055 (15)	−0.0041 (14)	−0.0044 (14)
C62	0.0249 (17)	0.0287 (16)	0.0240 (16)	−0.0053 (13)	0.0027 (14)	−0.0045 (13)
C61	0.0215 (16)	0.0184 (13)	0.0165 (14)	−0.0042 (12)	0.0039 (13)	−0.0044 (11)
C68	0.0295 (18)	0.0224 (15)	0.0284 (17)	−0.0077 (13)	0.0105 (15)	−0.0067 (13)
C63	0.0310 (19)	0.0306 (16)	0.0243 (17)	−0.0098 (14)	0.0059 (15)	0.0051 (14)
C49	0.039 (2)	0.049 (2)	0.0244 (18)	−0.0194 (17)	0.0098 (16)	−0.0122 (16)
C7	0.0331 (18)	0.0184 (14)	0.0234 (16)	−0.0075 (13)	−0.0010 (15)	−0.0032 (12)
C21`	0.0349 (19)	0.0234 (15)	0.0265 (17)	−0.0026 (14)	0.0031 (15)	−0.0021 (13)
C6	0.0299 (18)	0.0271 (16)	0.0223 (16)	−0.0072 (14)	0.0008 (14)	0.0003 (13)
C9	0.0313 (19)	0.0280 (16)	0.0286 (18)	−0.0014 (14)	−0.0051 (16)	−0.0006 (14)
C28	0.0330 (19)	0.0289 (16)	0.0220 (16)	−0.0107 (14)	−0.0020 (15)	0.0024 (13)
C31	0.038 (2)	0.0269 (16)	0.0247 (17)	0.0009 (15)	0.0033 (16)	−0.0016 (14)
C70	0.048 (2)	0.0230 (16)	0.0299 (19)	−0.0055 (16)	0.0172 (17)	−0.0001 (14)
C5	0.039 (2)	0.0265 (16)	0.0232 (16)	−0.0024 (15)	0.0042 (15)	−0.0010 (13)
C51	0.058 (3)	0.043 (2)	0.0226 (18)	−0.0116 (19)	−0.0083 (18)	0.0052 (16)
C45	0.038 (2)	0.0241 (17)	0.045 (2)	0.0039 (15)	0.0029 (18)	0.0065 (16)
C10	0.040 (2)	0.0284 (17)	0.0262 (17)	−0.0099 (15)	−0.0024 (16)	0.0001 (14)
C71	0.047 (2)	0.0256 (16)	0.0217 (17)	−0.0023 (16)	0.0080 (16)	−0.0010 (14)
C43	0.037 (2)	0.044 (2)	0.0286 (19)	−0.0104 (17)	0.0058 (17)	0.0044 (16)
C50	0.063 (3)	0.050 (2)	0.0233 (18)	−0.029 (2)	0.0089 (19)	−0.0039 (17)
C11	0.034 (2)	0.0344 (17)	0.0294 (18)	−0.0136 (15)	0.0081 (16)	−0.0079 (15)
C52	0.038 (2)	0.0333 (17)	0.0213 (17)	−0.0047 (15)	−0.0045 (15)	−0.0010 (14)
C64	0.036 (2)	0.0363 (18)	0.0195 (16)	−0.0019 (15)	0.0002 (15)	0.0031 (14)
C27	0.0296 (18)	0.0249 (15)	0.0188 (15)	−0.0069 (14)	0.0007 (14)	0.0024 (13)
C30	0.053 (2)	0.0346 (18)	0.0213 (17)	−0.0129 (17)	0.0065 (17)	−0.0098 (14)
C32	0.0335 (19)	0.0340 (17)	0.0209 (16)	−0.0086 (15)	0.0015 (15)	−0.0052 (14)
C25	0.052 (3)	0.051 (2)	0.036 (2)	−0.008 (2)	−0.014 (2)	0.0057 (19)
C24	0.054 (3)	0.050 (2)	0.0279 (19)	−0.001 (2)	0.0039 (19)	0.0095 (17)
C12	0.0298 (18)	0.0220 (15)	0.0334 (19)	−0.0023 (14)	−0.0024 (16)	−0.0017 (14)
C69	0.0304 (18)	0.0249 (16)	0.040 (2)	−0.0068 (14)	0.0121 (16)	−0.0107 (15)
C2	0.0319 (19)	0.0275 (16)	0.0306 (18)	−0.0090 (14)	−0.0019 (16)	0.0052 (14)
C1	0.0348 (19)	0.0204 (15)	0.0203 (16)	−0.0068 (14)	0.0042 (15)	0.0017 (13)
C29	0.043 (2)	0.0389 (18)	0.0201 (16)	−0.0192 (17)	−0.0004 (16)	−0.0026 (14)
C3	0.043 (2)	0.0322 (17)	0.0275 (18)	−0.0162 (16)	−0.0073 (17)	0.0026 (15)
C8	0.0302 (18)	0.0246 (15)	0.0282 (17)	−0.0046 (14)	0.0054 (15)	−0.0048 (14)
C22	0.036 (2)	0.045 (2)	0.034 (2)	−0.0106 (17)	−0.0005 (17)	−0.0026 (16)
C4	0.057 (2)	0.0277 (17)	0.0198 (16)	−0.0124 (17)	0.0004 (17)	−0.0028 (14)
C26	0.050 (2)	0.041 (2)	0.038 (2)	−0.0175 (18)	−0.0076 (19)	0.0041 (17)
C44	0.044 (2)	0.0288 (17)	0.039 (2)	−0.0046 (16)	0.0024 (18)	0.0091 (16)
C23	0.052 (3)	0.048 (2)	0.041 (2)	−0.0168 (19)	0.005 (2)	0.0073 (18)

### Geometric parameters (Å, °)

**Table:**

P1—O2	1.492 (2)	C63—C64	1.387 (4)
P1—O1	1.517 (2)	C63—H63	0.9500
P1—O3	1.608 (2)	C49—C50	1.367 (5)
P1—C1	1.780 (3)	C49—H49	0.9500
P2—O5	1.473 (3)	C7—C8	1.395 (4)
P2—O4	1.543 (2)	C7—C12	1.397 (4)
P2—O3	1.598 (2)	C21`—C22	1.376 (5)
P2—C7	1.773 (3)	C21`—C26	1.391 (5)
P21—O22	1.472 (3)	C6—C1	1.391 (4)
P21—O21	1.544 (3)	C6—C5	1.393 (4)
P21—O23	1.598 (2)	C6—H6	0.9500
P21—C21`	1.790 (3)	C9—C10	1.377 (5)
P22—O25	1.485 (2)	C9—C8	1.391 (4)
P22—O24	1.524 (3)	C9—H9	0.9500
P22—O23	1.608 (2)	C28—C29	1.385 (4)
P22—C27	1.775 (3)	C28—C27	1.390 (4)
P41—O42	1.482 (2)	C28—H28	0.9500
P41—O41	1.532 (2)	C31—C30	1.387 (5)
P41—O43	1.606 (2)	C31—C32	1.390 (4)
P41—C41	1.776 (3)	C31—H31	0.9500
P42—O45	1.478 (2)	C70—C71	1.365 (5)
P42—O44	1.531 (2)	C70—C69	1.392 (5)
P42—O43	1.606 (2)	C70—H70	0.9500
P42—C47	1.767 (3)	C5—C4	1.394 (5)
P62—O65	1.479 (2)	C5—H5	0.9500
P62—O64	1.532 (2)	C51—C50	1.387 (5)
P62—O63	1.6101 (19)	C51—C52	1.388 (5)
P62—C67	1.770 (3)	C51—H51	0.9500
P61—O62	1.482 (2)	C45—C44	1.370 (5)
P61—O61	1.540 (2)	C45—H45	0.9500
P61—O63	1.6049 (19)	C10—C11	1.395 (5)
P61—C61	1.780 (3)	C10—H10	0.9500
O41—H41	0.8400	C71—H71	0.9500
O64—H64	0.8400	C43—C44	1.388 (5)
O61—H61	0.8400	C43—H43	0.9500
O44—H44	0.8400	C50—H50	0.9500
O4—H4	0.8400	C11—C12	1.377 (4)
O21—H21	0.8400	C11—H11	0.9500
O24—H24	0.8400	C52—H52	0.9500
O1—H1	0.8400	C64—H64A	0.9500
C46—C45	1.385 (4)	C27—C32	1.396 (4)
C46—C41	1.400 (4)	C30—C29	1.387 (5)
C46—H46	0.9500	C30—H30	0.9500
C66—C65	1.388 (4)	C32—H32	0.9500
C66—C61	1.396 (4)	C25—C26	1.372 (5)
C66—H66	0.9500	C25—C24	1.373 (5)
C67—C68	1.401 (4)	C25—H25	0.9500
C67—C72	1.402 (4)	C24—C23	1.383 (5)
C48—C49	1.381 (4)	C24—H24A	0.9500
C48—C47	1.399 (4)	C12—H12	0.9500
C48—H48	0.9500	C69—H69	0.9500
C42—C43	1.385 (4)	C2—C3	1.383 (4)
C42—C41	1.391 (4)	C2—C1	1.390 (4)
C42—H42	0.9500	C2—H2	0.9500
C72—C71	1.393 (4)	C29—H29	0.9500
C72—H72	0.9500	C3—C4	1.384 (5)
C47—C52	1.388 (4)	C3—H3	0.9500
C65—C64	1.382 (5)	C8—H8	0.9500
C65—H65	0.9500	C22—C23	1.377 (5)
C62—C63	1.377 (4)	C22—H22	0.9500
C62—C61	1.393 (4)	C4—H4A	0.9500
C62—H62	0.9500	C26—H26	0.9500
C68—C69	1.380 (4)	C44—H44A	0.9500
C68—H68	0.9500	C23—H23	0.9500
			
O2—P1—O1	114.15 (14)	C8—C7—C12	120.0 (3)
O2—P1—O3	111.31 (14)	C8—C7—P2	120.3 (2)
O1—P1—O3	108.61 (13)	C12—C7—P2	119.7 (2)
O2—P1—C1	111.27 (14)	C22—C21`—C26	120.1 (3)
O1—P1—C1	109.97 (15)	C22—C21`—P21	117.7 (3)
O3—P1—C1	100.69 (13)	C26—C21`—P21	122.1 (3)
O5—P2—O4	117.45 (16)	C1—C6—C5	120.4 (3)
O5—P2—O3	113.31 (14)	C1—C6—H6	119.8
O4—P2—O3	101.76 (14)	C5—C6—H6	119.8
O5—P2—C7	110.02 (15)	C10—C9—C8	120.5 (3)
O4—P2—C7	109.34 (14)	C10—C9—H9	119.7
O3—P2—C7	103.89 (13)	C8—C9—H9	119.7
O22—P21—O21	116.66 (17)	C29—C28—C27	120.3 (3)
O22—P21—O23	114.18 (15)	C29—C28—H28	119.9
O21—P21—O23	101.71 (14)	C27—C28—H28	119.9
O22—P21—C21`	110.40 (16)	C30—C31—C32	120.0 (3)
O21—P21—C21`	109.76 (14)	C30—C31—H31	120.0
O23—P21—C21`	103.02 (14)	C32—C31—H31	120.0
O25—P22—O24	115.00 (15)	C71—C70—C69	121.2 (3)
O25—P22—O23	111.24 (14)	C71—C70—H70	119.4
O24—P22—O23	108.06 (13)	C69—C70—H70	119.4
O25—P22—C27	110.61 (13)	C6—C5—C4	118.9 (3)
O24—P22—C27	109.15 (15)	C6—C5—H5	120.5
O23—P22—C27	101.96 (13)	C4—C5—H5	120.5
O42—P41—O41	113.20 (12)	C50—C51—C52	119.8 (3)
O42—P41—O43	111.81 (12)	C50—C51—H51	120.1
O41—P41—O43	102.90 (11)	C52—C51—H51	120.1
O42—P41—C41	110.80 (13)	C44—C45—C46	120.4 (3)
O41—P41—C41	110.44 (14)	C44—C45—H45	119.8
O43—P41—C41	107.30 (12)	C46—C45—H45	119.8
O45—P42—O44	117.30 (13)	C9—C10—C11	120.0 (3)
O45—P42—O43	110.83 (11)	C9—C10—H10	120.0
O44—P42—O43	103.69 (11)	C11—C10—H10	120.0
O45—P42—C47	113.03 (13)	C70—C71—C72	119.9 (3)
O44—P42—C47	105.64 (13)	C70—C71—H71	120.1
O43—P42—C47	105.29 (13)	C72—C71—H71	120.1
O65—P62—O64	117.28 (13)	C42—C43—C44	119.9 (3)
O65—P62—O63	110.48 (11)	C42—C43—H43	120.0
O64—P62—O63	103.31 (11)	C44—C43—H43	120.0
O65—P62—C67	113.19 (13)	C49—C50—C51	120.5 (3)
O64—P62—C67	106.07 (13)	C49—C50—H50	119.8
O63—P62—C67	105.44 (12)	C51—C50—H50	119.8
O62—P61—O61	113.12 (11)	C12—C11—C10	120.2 (3)
O62—P61—O63	112.18 (11)	C12—C11—H11	119.9
O61—P61—O63	103.07 (11)	C10—C11—H11	119.9
O62—P61—C61	111.08 (13)	C47—C52—C51	120.0 (3)
O61—P61—C61	110.42 (13)	C47—C52—H52	120.0
O63—P61—C61	106.52 (11)	C51—C52—H52	120.0
P41—O41—H41	109.5	C65—C64—C63	120.3 (3)
P62—O64—H64	109.5	C65—C64—H64A	119.9
P61—O63—P62	130.24 (13)	C63—C64—H64A	119.9
P41—O43—P42	130.60 (12)	C28—C27—C32	119.5 (3)
P61—O61—H61	109.5	C28—C27—P22	120.8 (2)
P42—O44—H44	109.5	C32—C27—P22	119.7 (2)
P2—O4—H4	109.5	C29—C30—C31	120.1 (3)
P2—O3—P1	131.31 (15)	C29—C30—H30	120.0
P21—O21—H21	109.5	C31—C30—H30	120.0
P21—O23—P22	130.90 (15)	C31—C32—C27	120.0 (3)
P22—O24—H24	109.5	C31—C32—H32	120.0
P1—O1—H1	109.5	C27—C32—H32	120.0
C45—C46—C41	119.7 (3)	C26—C25—C24	120.9 (4)
C45—C46—H46	120.2	C26—C25—H25	119.6
C41—C46—H46	120.2	C24—C25—H25	119.6
C65—C66—C61	119.3 (3)	C25—C24—C23	119.6 (3)
C65—C66—H66	120.4	C25—C24—H24A	120.2
C61—C66—H66	120.4	C23—C24—H24A	120.2
C68—C67—C72	119.4 (3)	C11—C12—C7	119.9 (3)
C68—C67—P62	120.3 (2)	C11—C12—H12	120.0
C72—C67—P62	120.3 (2)	C7—C12—H12	120.0
C49—C48—C47	120.0 (3)	C68—C69—C70	119.7 (3)
C49—C48—H48	120.0	C68—C69—H69	120.2
C47—C48—H48	120.0	C70—C69—H69	120.2
C43—C42—C41	119.9 (3)	C3—C2—C1	120.1 (3)
C43—C42—H42	120.0	C3—C2—H2	120.0
C41—C42—H42	120.0	C1—C2—H2	120.0
C42—C41—C46	119.6 (3)	C2—C1—C6	119.8 (3)
C42—C41—P41	121.0 (2)	C2—C1—P1	120.5 (2)
C46—C41—P41	119.4 (2)	C6—C1—P1	119.6 (2)
C71—C72—C67	119.8 (3)	C28—C29—C30	120.1 (3)
C71—C72—H72	120.1	C28—C29—H29	119.9
C67—C72—H72	120.1	C30—C29—H29	119.9
C52—C47—C48	119.4 (3)	C2—C3—C4	120.0 (3)
C52—C47—P42	120.5 (2)	C2—C3—H3	120.0
C48—C47—P42	120.1 (2)	C4—C3—H3	120.0
C64—C65—C66	120.4 (3)	C9—C8—C7	119.4 (3)
C64—C65—H65	119.8	C9—C8—H8	120.3
C66—C65—H65	119.8	C7—C8—H8	120.3
C63—C62—C61	120.4 (3)	C21`—C22—C23	119.9 (3)
C63—C62—H62	119.8	C21`—C22—H22	120.1
C61—C62—H62	119.8	C23—C22—H22	120.1
C62—C61—C66	119.8 (3)	C3—C4—C5	120.7 (3)
C62—C61—P61	120.4 (2)	C3—C4—H4A	119.6
C66—C61—P61	119.8 (2)	C5—C4—H4A	119.6
C69—C68—C67	120.0 (3)	C25—C26—C21`	119.3 (4)
C69—C68—H68	120.0	C25—C26—H26	120.3
C67—C68—H68	120.0	C21`—C26—H26	120.3
C62—C63—C64	119.8 (3)	C45—C44—C43	120.4 (3)
C62—C63—H63	120.1	C45—C44—H44A	119.8
C64—C63—H63	120.1	C43—C44—H44A	119.8
C50—C49—C48	120.4 (3)	C22—C23—C24	120.2 (4)
C50—C49—H49	119.8	C22—C23—H23	119.9
C48—C49—H49	119.8	C24—C23—H23	119.9
			
O62—P61—O63—P62	−77.15 (18)	O5—P2—C7—C12	−18.7 (3)
O61—P61—O63—P62	160.86 (16)	O4—P2—C7—C12	−149.0 (2)
C61—P61—O63—P62	44.6 (2)	O3—P2—C7—C12	102.9 (3)
O65—P62—O63—P61	39.8 (2)	O22—P21—C21`—C22	−36.8 (3)
O64—P62—O63—P61	166.04 (16)	O21—P21—C21`—C22	93.2 (3)
C67—P62—O63—P61	−82.84 (19)	O23—P21—C21`—C22	−159.1 (3)
O42—P41—O43—P42	−78.49 (19)	O22—P21—C21`—C26	147.2 (3)
O41—P41—O43—P42	159.72 (16)	O21—P21—C21`—C26	−82.8 (3)
C41—P41—O43—P42	43.2 (2)	O23—P21—C21`—C26	24.9 (3)
O45—P42—O43—P41	36.2 (2)	C1—C6—C5—C4	0.5 (5)
O44—P42—O43—P41	162.96 (16)	C41—C46—C45—C44	1.7 (5)
C47—P42—O43—P41	−86.29 (19)	C8—C9—C10—C11	−0.1 (5)
O5—P2—O3—P1	−73.7 (2)	C69—C70—C71—C72	0.1 (5)
O4—P2—O3—P1	53.4 (2)	C67—C72—C71—C70	0.1 (5)
C7—P2—O3—P1	166.9 (2)	C41—C42—C43—C44	−0.2 (5)
O2—P1—O3—P2	34.0 (2)	C48—C49—C50—C51	0.5 (5)
O1—P1—O3—P2	−92.5 (2)	C52—C51—C50—C49	−0.2 (5)
C1—P1—O3—P2	152.0 (2)	C9—C10—C11—C12	0.6 (5)
O22—P21—O23—P22	71.7 (3)	C48—C47—C52—C51	−0.6 (5)
O21—P21—O23—P22	−54.9 (2)	P42—C47—C52—C51	179.9 (3)
C21`—P21—O23—P22	−168.6 (2)	C50—C51—C52—C47	0.3 (5)
O25—P22—O23—P21	−26.3 (3)	C66—C65—C64—C63	−0.2 (5)
O24—P22—O23—P21	100.8 (2)	C62—C63—C64—C65	0.8 (5)
C27—P22—O23—P21	−144.3 (2)	C29—C28—C27—C32	−0.3 (5)
O65—P62—C67—C68	−179.3 (2)	C29—C28—C27—P22	177.9 (2)
O64—P62—C67—C68	50.8 (3)	O25—P22—C27—C28	162.1 (2)
O63—P62—C67—C68	−58.4 (3)	O24—P22—C27—C28	34.6 (3)
O65—P62—C67—C72	−1.8 (3)	O23—P22—C27—C28	−79.5 (3)
O64—P62—C67—C72	−131.8 (2)	O25—P22—C27—C32	−19.7 (3)
O63—P62—C67—C72	119.1 (2)	O24—P22—C27—C32	−147.2 (2)
C43—C42—C41—C46	1.0 (5)	O23—P22—C27—C32	98.7 (3)
C43—C42—C41—P41	−179.1 (3)	C32—C31—C30—C29	0.4 (5)
C45—C46—C41—C42	−1.7 (5)	C30—C31—C32—C27	−0.7 (5)
C45—C46—C41—P41	178.4 (3)	C28—C27—C32—C31	0.7 (5)
O42—P41—C41—C42	−162.5 (2)	P22—C27—C32—C31	−177.5 (2)
O41—P41—C41—C42	−36.3 (3)	C26—C25—C24—C23	0.8 (6)
O43—P41—C41—C42	75.1 (3)	C10—C11—C12—C7	0.0 (5)
O42—P41—C41—C46	17.4 (3)	C8—C7—C12—C11	−1.1 (4)
O41—P41—C41—C46	143.6 (2)	P2—C7—C12—C11	176.7 (2)
O43—P41—C41—C46	−105.0 (3)	C67—C68—C69—C70	0.7 (4)
C68—C67—C72—C71	0.1 (4)	C71—C70—C69—C68	−0.5 (5)
P62—C67—C72—C71	−177.4 (2)	C3—C2—C1—C6	0.3 (5)
C49—C48—C47—C52	0.9 (5)	C3—C2—C1—P1	−175.6 (2)
C49—C48—C47—P42	−179.6 (2)	C5—C6—C1—C2	−1.1 (5)
O45—P42—C47—C52	0.2 (3)	C5—C6—C1—P1	174.8 (2)
O44—P42—C47—C52	−129.3 (3)	O2—P1—C1—C2	−162.5 (2)
O43—P42—C47—C52	121.3 (3)	O1—P1—C1—C2	−35.1 (3)
O45—P42—C47—C48	−179.3 (2)	O3—P1—C1—C2	79.4 (3)
O44—P42—C47—C48	51.2 (3)	O2—P1—C1—C6	21.6 (3)
O43—P42—C47—C48	−58.2 (3)	O1—P1—C1—C6	149.1 (2)
C61—C66—C65—C64	−0.5 (4)	O3—P1—C1—C6	−96.4 (3)
C63—C62—C61—C66	0.0 (4)	C27—C28—C29—C30	0.0 (5)
C63—C62—C61—P61	−178.7 (2)	C31—C30—C29—C28	0.0 (5)
C65—C66—C61—C62	0.6 (4)	C1—C2—C3—C4	1.1 (5)
C65—C66—C61—P61	179.3 (2)	C10—C9—C8—C7	−1.1 (5)
O62—P61—C61—C62	−163.0 (2)	C12—C7—C8—C9	1.6 (4)
O61—P61—C61—C62	−36.6 (3)	P2—C7—C8—C9	−176.2 (2)
O63—P61—C61—C62	74.6 (2)	C26—C21`—C22—C23	−0.1 (5)
O62—P61—C61—C66	18.3 (3)	P21—C21`—C22—C23	−176.1 (3)
O61—P61—C61—C66	144.7 (2)	C2—C3—C4—C5	−1.7 (5)
O63—P61—C61—C66	−104.1 (2)	C6—C5—C4—C3	0.9 (5)
C72—C67—C68—C69	−0.5 (4)	C24—C25—C26—C21`	−1.5 (6)
P62—C67—C68—C69	177.0 (2)	C22—C21`—C26—C25	1.1 (5)
C61—C62—C63—C64	−0.7 (4)	P21—C21`—C26—C25	177.0 (3)
C47—C48—C49—C50	−0.8 (5)	C46—C45—C44—C43	−0.9 (5)
O5—P2—C7—C8	159.2 (2)	C42—C43—C44—C45	0.1 (5)
O4—P2—C7—C8	28.8 (3)	C21`—C22—C23—C24	−0.6 (6)
O3—P2—C7—C8	−79.2 (3)	C25—C24—C23—C22	0.3 (6)

### Hydrogen-bond geometry (Å, °)

**Table:**

*D*—H···*A*	*D*—H	H···*A*	*D*···*A*	*D*—H···*A*
O41—H41···O45^i^	0.84	1.69	2.530 (3)	175
O64—H64···O62^ii^	0.84	1.67	2.488 (3)	163
O61—H61···O65^iii^	0.84	1.72	2.551 (3)	171
O4—H4···O25	0.84	1.69	2.464 (3)	153
O21—H21···O2	0.84	1.70	2.472 (3)	152
O24—H24···O22^i^	0.84	1.67	2.464 (3)	156
O1—H1···O5^iii^	0.84	1.66	2.456 (3)	157

Symmetry codes: (i) *x*−1, *y*, *z*; (ii) −*x*+1, −*y*, −*z*+1; (iii) *x*+1, *y*, *z*.
